# Can gamified surgical sets improve surgical instrument recognition and student performance retention in the operating room? A multi-institutional experimental crossover study

**DOI:** 10.1186/s12909-023-04868-z

**Published:** 2023-11-29

**Authors:** Mohsen Masoumian Hosseini, Zahra Sadat Manzari, Azam Gazerani, Seyedeh Toktam Masoumian Hosseini, Akram Gazerani, Mehrdad Rohaninasab

**Affiliations:** 1 Department of E-Learning in Medical Science, Tehran University of Medical Sciences, Tehran, Iran; 2 CyberPatient Research Affiliate, Interactive Health International, Department of Surgery, University of British Columbia, Vancouver, Canada; 3 Nursing and Midwifery Care Research Center, Mashhad University of Medical Sciences, Mashhad, Iran; 4 Department of Nursing, Neyshabur University of Medical Sciences, Neyshabur, Iran; 5 Department of Nursing, School of Nursing and Midwifery, Torbat Heydariyeh University of Medical Sciences, Torbat Heydariyeh, Iran; 6 Student Research Committee, School of Nursing and Midwifery, Mashhad University of Medical Sciences, Mashhad, Iran; 7 Department of Operating Room, Neyshabur University of Medical Sciences, Neyshabur, Iran

**Keywords:** Game, Crossover, Operating room, Surgical sets, Surgical instrument, Surgical technology students, Traditional teaching, OSCE, Performance retention

## Abstract

**Introduction:**

Surgery requires a high degree of precision, speed, and concentration. Owing to the complexity of the modern world, traditional methods cannot meet these requirements. Therefore, in this study, we investigated students’ diagnostic skills in the Operating Room in the context of surgical instruments by using gamification of surgical instruments and a crossover design.

**Method:**

The study design was a multi-institutional quasi-experimental crossover and involved a three-arm intervention (with gender-specific block randomisation: Group A, B, and C) with a pre-test and three post-tests. A total of 90 students fell into three groups of 30 participants each. The surgical sets were learned for one semester through game-based instruction and traditional teaching, and then three OSCE tests were administered with time and location differences. Using one-way ANOVA, OSCE results were compared in the game, traditional, and control groups. The effectiveness of the intervention was tested in each group by repeated measures.

**Result:**

The pretest scores of all three groups did not differ significantly. In the OSCE tests, both groups, A and B, performed similarly. However, these tests showed a significant difference in grouping between training through games and training in the traditional way. There was no significant difference between OSCE tests 2 and 3 in the game-based training group, indicating that what was learned was retained, while in the traditional method training group, OSCE 3 test scores declined significantly. Furthermore, repeated measures showed the effectiveness of game-based training.

**Conclusion:**

In this study, gamification has turned out to be very effective in helping learners learn practical skills and leading to more sustainable learning.

## Introduction

Students and staff in the operating room are constantly confronted with new surgical procedures and changing technologies that make working in the operating room challenging and complex [1]. They need more skills to deliver quality care to their clients and make quick and accurate decisions in critical situations. So, the first mistake in surgery can also be the last mistake that leaves irreparable damage to the patient [2]. The bachelor’s program in surgical technology is an emerging field in Iran that requires practical and clinical skills. This course is intended for professionals who will join the surgical team and play an essential role in delivering a successful operation. The surgical technology students and mobile staff are essential to complete the surgery, and their training is only possible if they can work in an actual surgical environment [3]. Therefore, clinical training and competence are essential to the surgical profession.

Professional activity in clinical environments demands the acquisition of a multitude of competencies. To enhance the educational experience, it is more effective to opt for competency-based education instead of traditional approaches [4]. This approach leverages technology to facilitate maximum learning outcomes, emphasizing the critical importance of integrating technology into training programs [5].

Today’s generation of students, often referred to as digital natives, have seamlessly integrated technology into every aspect of their lives [6]. They rely heavily on electronic tools and resources to enhance their learning experiences and acquire knowledge dynamically and interactively. Traditional educational approaches, however, are failing to meet the unique needs and preferences of these digitally savvy learners [7]. In order for education to remain relevant and effective in the 21st century, it is imperative that universities adapt and embrace the potential offered by evolving technologies [8]. This means incorporating digital platforms like interactive games [9], virtual simulators [10], smart devices like wearable tech [11], or social media channels into classroom instruction. By doing so, educators can tap into students’ inherent motivation stemming from their familiarity with technology [7]. Neglecting this need for technological integration may result in a decline in popularity among traditional education systems. Students today thrive with access to engaging digital resources that cater specifically to their learning styles [12]. It is essential for formal education institutions to not only recognize but also fully utilize the opportunities presented by advancing technologies if they wish to provide truly effective learning experiences for future generations [13].

One of the biggest challenges in training students in the operating room is tantamount to training qualified students who can work effectively in a clinical setting. At the same time, staff and managers are worried about the performance of new graduates [14]. Traditional training has lost its effectiveness in the modern world because it is time and place-bound and cannot provide an accurate and relevant learning context [15]. However, translating theoretical knowledge into psychomotor skills is complex and challenges the teaching process in clinical settings [16]. By playing computer games, students can apply their knowledge and gain valuable experience in the virtual world that will shape their future behaviour [9]. Game-based learning is one of the active learning methods. The two main aspects of the gamification system are that learning is fun and challenging for learners, which increases engagement and motivation. Part of the system also allows learners to experiment and, in addition to the challenge, permits them to analyse their level of performance and progress [17].

Learning games are an appropriate and necessary means of discovering and developing information resources to prepare learners to acquire the necessary skills. Learning and recognising surgical instruments and their placement are essential skills for students in the operating room [18]. They contribute to surgeon satisfaction by reducing complications during anaesthesia, speeding up surgery and shortening the duration of surgery. For this reason, this study considered utilising the game as an arrangement of surgical sets to measure student performance in a clinical setting.

## Material and method

**Fig. 1:**
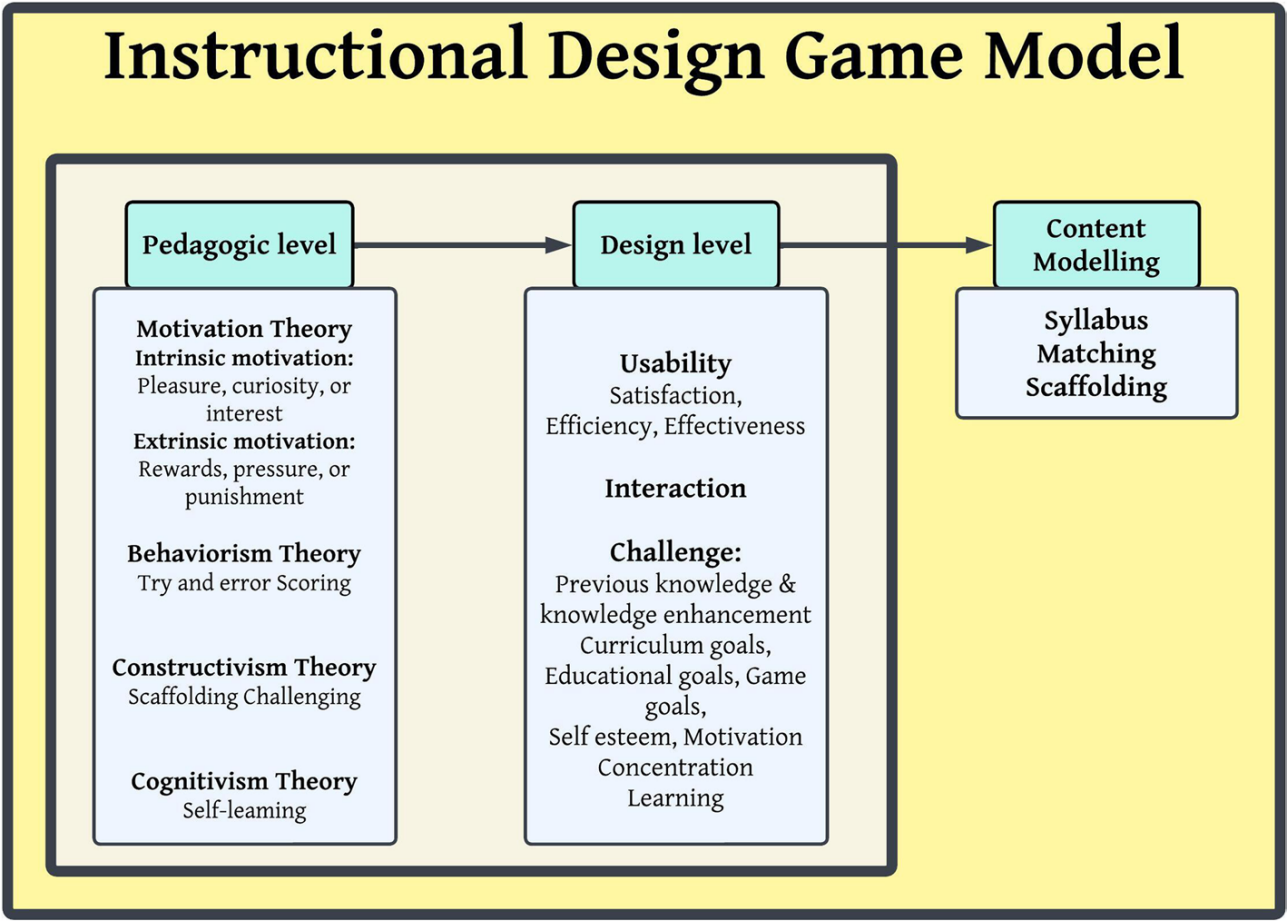
Game development educational framework

The game was developed based on the proposed educational design model in Fig. 1. After the necessary assessments were carried out, the intervention was implemented to teach how to arrange surgical sets.

### Study design

**Fig. 2:**
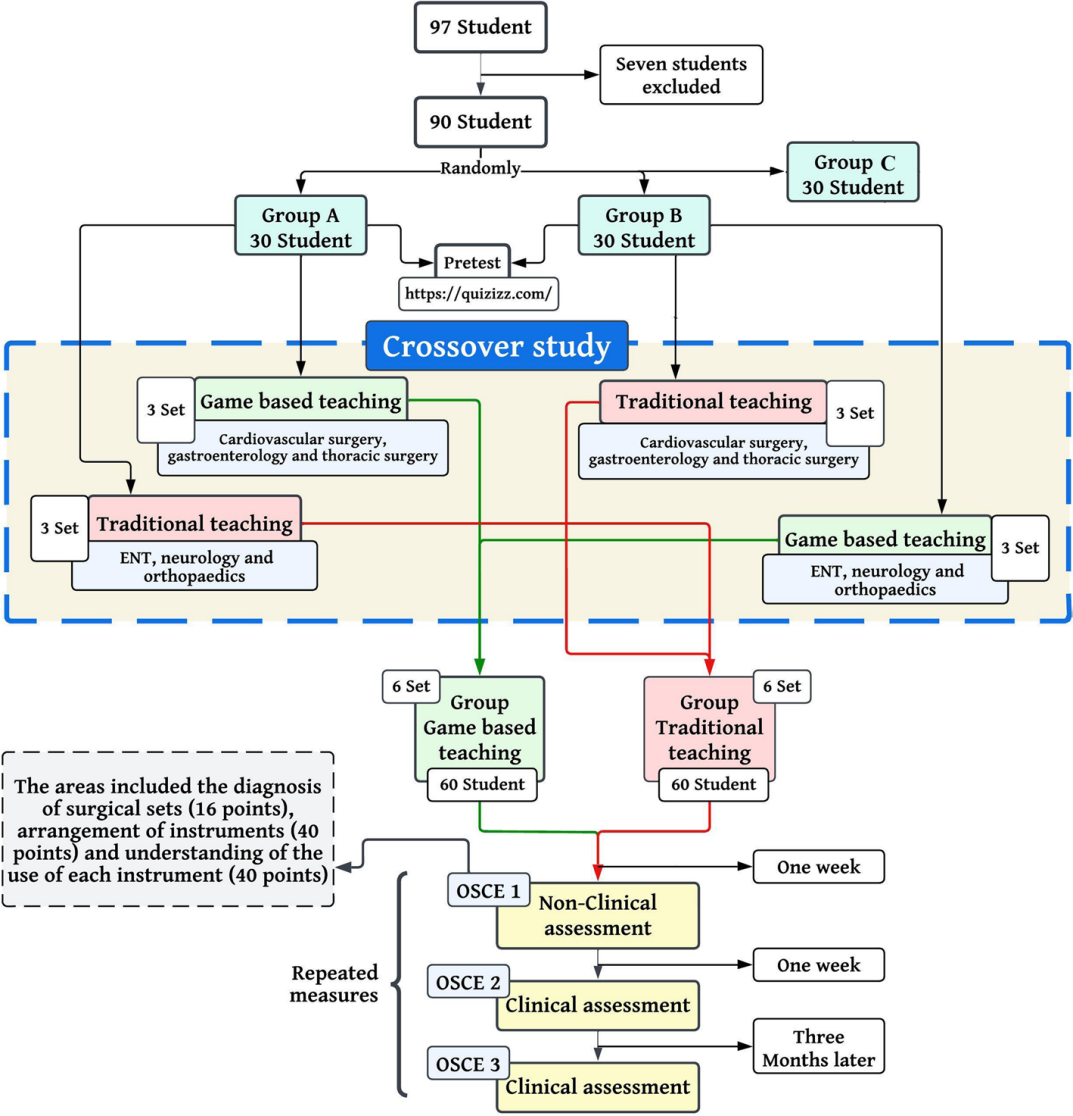
An overview of the study process and the crossover design for the intervention arm

The study design was a multi-institutional experimental crossover with an intervention arm (gamification ∞ traditional) and a control arm and included a pre-test and three post-tests. An investigation of the effects of the gamified surgical set on performance change and sustainability was conducted at three time points and three sites. Figure 2 shows the flowchart of the study design.

### Game design

This study used articulate Storyline 3, Adobe Premiere Pro and Adobe Photoshop to create the game. Adobe Premiere Pro was used to create the logo introduction. Adobe Photoshop was used to design the images, and Articulate Storyline was used to design the main framework of the game. Articulate Storyline is one of the most popular programmes for designing interactive electronic content. Adobe Photoshop is a raster graphics editor developed by Adobe Inc. One of the best multimedia programmes from Adobe is Adobe Premiere Pro.

The game was established in Articulate Storyline software using the drag-and-drop method and composes of two parts, the teaching and the learning assessment. Educating part with six surgeries was divided into cardiology, gastrointestinal, thoracic, ENT, neurology and orthopaedics. The surgeries mentioned, only those scheduled for each group were presented, and the service team locked the rest. The most commonly used surgical instruments for each of these surgeries were selected and assembled. General surgery sets included appendectomy, laparotomy, inguinal hernia, haemorrhoids and open cholecystectomy. Orthopaedic surgery sets included screw and plate fractures, cts, knee arthroscopy and dhs. Ophthalmic surgery sets included chalazion and pterygium. ENT surgery sets included myringotomy, tonsillectomy, tympanoplasty, rhinoplasty, sinus surgery, and maxillary hernia. Plastic surgery sets included skin graft and liposuction. Sets for neurosurgery included craniotomies, laminectomies and coronary bypasses. Ophthalmic surgery sets included DCR cataract, cornea and laparoscopy devices.

**Fig. 3:**
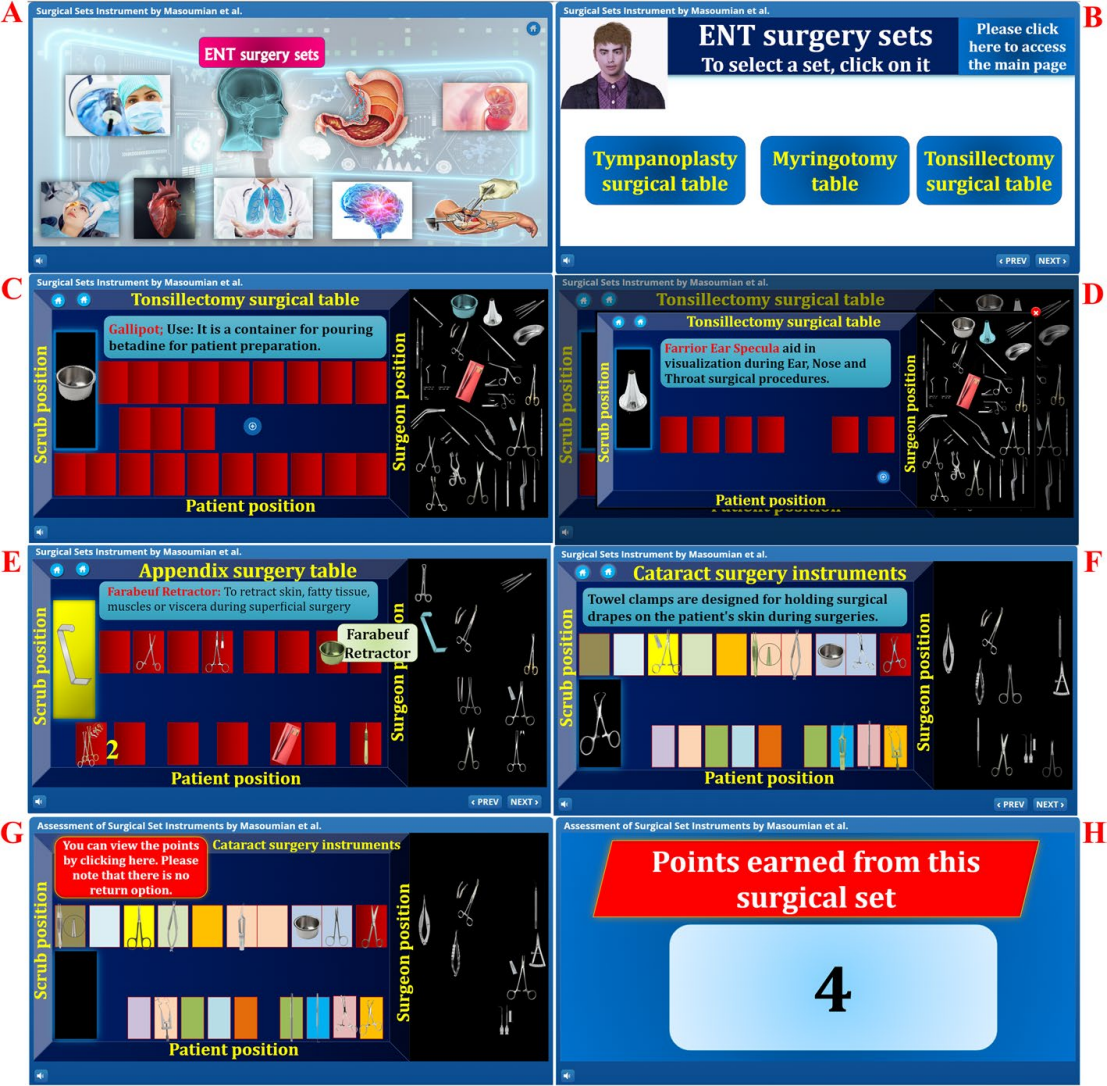
These pictures illustrate the game environment. **A** This section shows the student a selection of surgical sets available in this game in different categories. **B** In this section, the student has the option of selecting surgical sets for a particular surgical procedure. **C**, **D**, **E** and **F** The pictures illustrate the learning part of the game, where the student has to place each instrument in the correct position. If a mistake is made, the surgical instrument returns to its correct place in the menu. **G** and **H** The pictures illustrate the learning assessment section, in which students are allowed to place each instrument in one of the game boxes and only receive positive points if the instrument is in the correct position

Surgical sets were modelled on the shape of an operating table, so each slide contained a section for selecting instruments and an operating table (Fig. 3). A typical operating table has three sides: the patient, surgeon, and scrub. On this table, separate boxes were designed for each surgical instrument, depending on its position. The technical team was responsible for creating the content designed for the game so that instruments could be selected by dragging them from the surgical equipment and placed only in the appropriate boxes (Fig. 3, sections E and F). When hovering over a surgical instrument, a small tutorial appears at the top of the programme explaining the instrument and its use; for simplicity, this instrument is the larger one on the left side of the screen. The learning section teaches the student how to use each surgical instrument and its specific position within the surgical set (Fig. 3, sections A-F). The game assessment section was also created by the drag-and-drop method, with only three significant differences from the learning section: 1. In this section, students could place any surgical instrument in any box. 2. If the student selects the correct surgical instrument and puts it in the correct place in the game, they receive a positive score. 3. Three surgical tables were designed per surgery (Fig. 3 sections G and H). This game was produced in SCORM format and uploaded to the university’s Learning Management System (LMS). It then makes available to individual student groups.

### Sample and setting

Ad hoc analysis with G-Power software to achieve 84% statistical power (β = 0.17), estimating moderate primary outcome effect of 0.33, with the 2-sided test at α = 0.05, revealed a minimum of 29 participants were required for each intervention arm. Initially, 97 students were selected from the operating room department through a census. Students enrolled in the operating room department at NUMS = 64, and MUMS = 33 in 2021 were eligible to participate in the study. Seven students were excluded from the study (*N* = 90) because they were not computer literate (based on self-assessment), had the experience of working in an operating room, had prior knowledge of setting up an operating table and had attended relevant workshops in the last 6 months. An internet-based computer-generated random sequence was used to stratify students by gender and randomly assign them to three arms [19]. The participant groups were divided into three arms (A: gamified first three sets of surgery, B: gamified second three sets of surgery, and C: conventional surgical sets). A flowchart illustrating the participant recruitment process is shown in Fig. 2. First, the researcher visited the research site and described the aim of the study, which was to improve behavioural skills related to surgical tools. The students were also assured that their grades would not affect their internship course, that their data would remain confidential, and that only general data would be published. The study was conducted at three locations: the university’s LMS system, the practice room, and the hospital. Ethical codes were considered for each site.

### Pretraining intervention

**Fig. 4:**
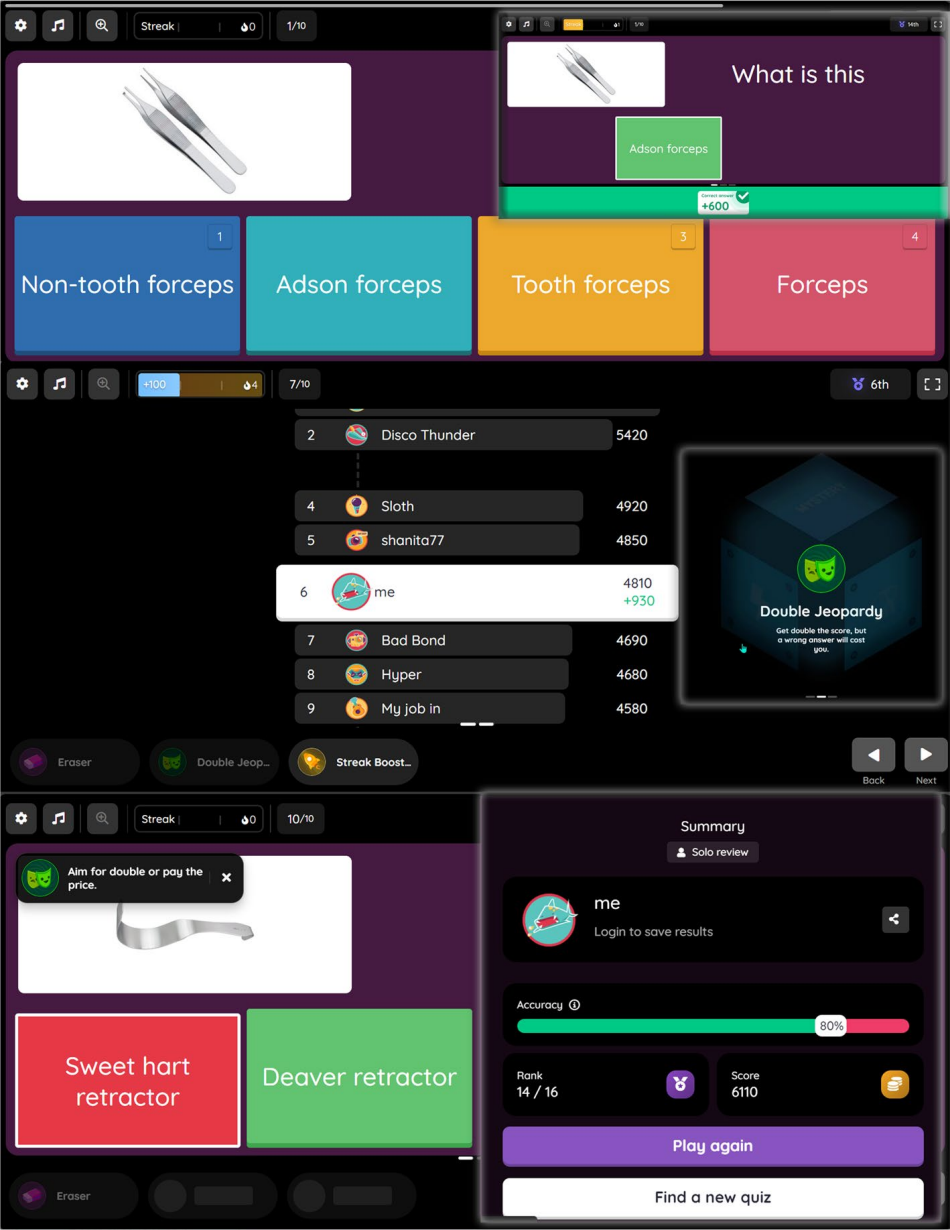
Pre-test execution using the website www.quizizz.com

Before the intervention, a pretest was conducted to assess the homogeneity of the performances of the three groups. The test consisted of 44 MSQ questions related to the performance and arrangement of surgical instrument sets. The test was administered via https://quizizz.com/, and the connection link was https://quizizz.com/join?gc=56768083. Each question was scored with one point. The link to the pre-test was also included in the student LMS. Seven faculty members from operating room confirmed the qualitative content validity of the test, and 10 students with characteristics similar to those of the target population confirmed its qualitative face validity. We conducted a test-retest analysis with a 14-day interval to assess the internal consistency and stability of the test. A total of 30 students from operating room, who were not to participate in the main analysis, took the test and obtained satisfactory results with the Kuder-Richardson 20 (KR20, *r* = 0.73) and the IntraClass Correlation (ICC, *r* = 0.83) [20]. A visual representation of the pre-test can be found in Fig. 4.

### Implementing intervention

According to the Ministry of Health and Medical Education programme in Iran, the course “Introduction to Surgical Instruments and Equipment” includes two theoretical credits equivalent to 17 2-h sessions. The corresponding professor presented the course in 17 theoretical sessions in the second semester. The same course plan, lesson plan, training approach and assessment method was used for the three groups’ students. In this course, the intervention was a part of the training programme and consisted of seven sessions followed by competency tests for the students.

At the beginning of the first phase of the intervention, which lasted 30 days and included 2 h of training per week, two teaching methods were used. Sets of 6 surgeries were designed for training the students. The sets for cardiovascular surgery, gastroenterology and thoracic surgery in Group A were taught using the game method, while three other sets, including ENT, neurology and orthopaedics, were taught traditionally. In contrast to group A, group B was traditionally taught the first three sets, and the rest sets through the game (crossover).

#### Traditional sessions

The conventional method held classes at 4-day intervals for a month. The teacher imparted the information while the students acted as semi-passive receivers. The conventional training method included using an LMS to teach the same topics regarding the names and applications of surgical equipment and instruments. Since the start of the 2019 coronavirus pandemic (COVID -19), this virtual training method has been routinely applied at most universities in Iran. Each session included 2 h of lecture, learning resources (such as multimedia, textbooks and podcasts), assignments and self-tests with multiple-choice questions. As for the theoretical sessions, the training was delivered through lectures in the LMS. The first day of the additional session was dedicated to the delivery of an online course based on lectures and Microsoft PowerPoint presentations, in which students were trained in the principles of arranging surgical instruments in minor general surgery, similar to the “virtual version” of the surgical instrument arrangement game. In all sessions, students were trained using the third edition of the book “Differentiating Surgical Instruments” [21]. A copy of the book was available in the LMS system. In addition, students were presented with a series of assignments and self-tests to complete independently during the 4-day break between sessions. In addition, students had the opportunity to communicate with their professor via the “message module” if needed.

#### Game-based learning sessions

The game-based training includes the game “Surgical instruments arrangement”. In order to run the game on the LMS platform, it was developed in Scorm format. A video clip was presented on the LMS as a first step to show how the game should be played. Students were instructed to play the game individually on the computer whenever they wanted for each week and to discuss the game and their results with their teacher and classmates formally online. To solve problems related to the game, students were also instructed to send messages to their professor via the “message module”. Students in the game could not use other LMS features (such as assignments and self-examinations).

In game-based learning sessions, all learning activities were based on surgical sets presented to the students in each session. Students were given feedback if they made an incorrect choice so that the surgical instrument returned to its original location if they made an incorrect placement. In addition, students were allowed to make mistakes during the assessment to create competition. They received a +1 plus point for correctly placed surgical instruments. At the end of each session, the results of each group and the overall results were displayed. As part of the project, the researcher developed virtual avatars and confidentially displayed each student’s score on a leaderboard in the university’s LMS so that students could compare their performance with that of their peers. During the game training, which was also virtual, the teacher acted as a facilitator, and the students were active and self-directed. Students interacted with each other during the game and gave feedback to their fellow students. This crossover study provided students in both groups with a combination of game and traditional training. For an overview of the study process, see Fig. 2.

#### Control group

Participants in the control group did not receive any game-based assessment or feedback. They were mandated to use the time between tasks to reflect and set their training goals. It is founded on the principles of experiential learning, which involves active experimentation and reflective observation and provides a basis for performance improvement, learning without feedback.

### Phase one: assessing performance in a non-clinical setting

The students were given 1 week to prepare after completing the intervention. Then, the OSCE test was conducted in a dedicated practice room at the university, creating an environment that closely resembled real-life surgical scenarios. This comprehensive assessment consisted of six distinct stations, each specifically designed to evaluate surgical skills. Four surgical sets were allocated for each type of surgery being tested to ensure thorough evaluation and accuracy. A panel of three experts meticulously assessed every student’s performance across three crucial domains within the test to provide an accurate assessment. The areas included the diagnosis of surgical sets (16 points), arrangement of instruments (40 points) and understanding of the use of each instrument (40 points). In addition, 1 point was awarded for each surgical set based on the time taken to complete it (24 points). In order to determine the exact time needed to complete each set, three experts were asked to complete each set in the shortest time possible; Their mean time was used to calculate the exact time. Table 1 shows the mean time required to complete each surgical set.

**Table 1:** The mean performance exact time of three experts in the arrangement of each surgical set

**Name of set**	**Number of instruments**	**Situation**	**Mean of time**
**General surgery sets**			
Haemorrhoid	28	Non-Clinical	3.21
		Clinical	3.42
Appendectomy	27	Non-Clinical	3.1
		Clinical	3.39
Laparotomy	61	Non-Clinical	6.99
		Clinical	7.38
Inguinal hernia	27	Non-Clinical	2.98
		Clinical	3.23
Cholecystectomy (laparoscopic surgery)	52	Non-Clinical	10.41
		Clinical	10.55
Cholecystectomy (open surgery)	64	Non-Clinical	7.42
		Clinical	7.62
**Orthopaedic surgery sets**			
Screw and plate fractures	37	Non-Clinical	4.24
		Clinical	4.27
Cts (Carpal Tunnel Syndrome)	21	Non-Clinical	2.46
		Clinical	2.55
Knee arthroscopy	39	Non-Clinical	4.47
		Clinical	4.86
DHS	13	Non-Clinical	1.49
		Clinical	1.56
**Ophthalmic surgery sets**			
Chalazion	22	Non-Clinical	2.52
		Clinical	2.61
DCR	43	Non-Clinical	5.02
		Clinical	5.20
Cataract	26	Non-Clinical	3.18
		Clinical	3.36
Pterygium	15	Non-Clinical	2.03
		Clinical	2.15
**ENT surgery sets**			
Myringotomy	18	Non-Clinical	2.06
		Clinical	2.1
Tonsillectomy	29	Non-Clinical	3.32
		Clinical	3.41
Tympanoplasty	48	Non-Clinical	5.49
		Clinical	5.62
Rhinoplasty	54	Non-Clinical	6.18
		Clinical	6.33
Orthognathic	56	Non-Clinical	6.41
		Clinical	6.59
Sinus surgery	56	Non-Clinical	6.41
		Clinical	6.62
**Plastic surgery sets**			
Skin graft	21	Non-Clinical	2.46
		Clinical	2.55
Liposuction	20	Non-Clinical	2.04
		Clinical	2.30
**Neurosurgery sets**			
Craniotomy	64	Non-Clinical	7.21
		Clinical	7.44
Laminectomies	60	Non-Clinical	6.87
		Clinical	7.11
Ventriculoperitoneal shunt	63	Non-Clinical	7.05
		Clinical	7.20
**Cardiovascular set**			
Coronary bypasses	57	Non-Clinical	10.05
		Clinical	10.40
Vascular general set	57	Non-Clinical	6.51
		Clinical	6.59
**Orology set**			
Nephrectomy	65	Non-Clinical	7.42
		Clinical	7.46
TUL	18	Non-Clinical	3.21
		Clinical	3.36
TURP	19	Non-Clinical	3.40
		Clinical	3.55
PCNL	35	Non-Clinical	5.03
		Clinical	5.21

The validity and reliability of the tests (pretest and posttest) were assessed by seven representatives of the nursing faculty and the operating room. In addition, inter-rater reliability was measured by comparing the ratings of 30 surgical technology students (who did not participate in the main analysis) with those of two operating room lecturers with similar professional characteristics. The ICC coefficients for the inter-faculty agreement were 0.82–0.91, indicating a high level of agreement between the assessments [22]. To eliminate bias, raters at each university who completed the OSCE form were trained by the principal investigator on how to evaluate student performance. The ICC coefficients for the inter-assessor agreement were 0.84–0.93.

### Phase two: assessing performance in a clinical setting

One week after the OSCE test, another similar test was administered to the students in the hospital operating room. However, the exact time of re-performance in the hospital was calculated for this test (Table 2).

**Table 2:**
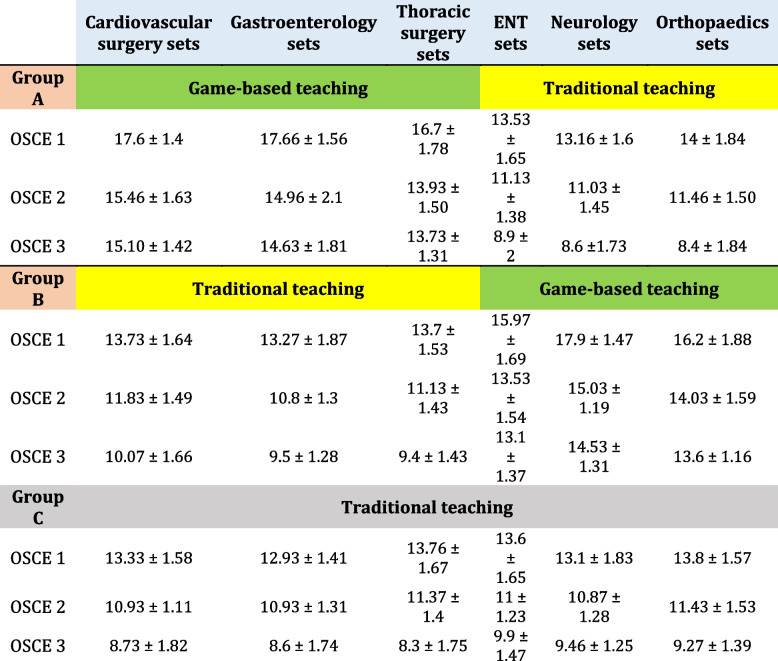
Comparison of the mean (±SD) performance of 3 groups in each of the six surgical sets of the OSCE exam (1, 2, and 3)

### Phase three: follow-up of performance (durability of performance)

Three months after the procedure, a test similar to the previous OSCE test was performed in the hospital to determine the durability of the performance.

### Data processing and analysis

In this study, SPSS version 26 was used to compile, enter and analyse the data. *Statistical significance* was defined as a *p*-value of less than 0.05. The Shapiro-Wilk test was used to assess the normality of the data distribution. Levene’s test was performed to test for equality of variance, and Tukey’s test for multiple comparisons was performed to ensure that the assumptions of analysis of variance (ANOVA) were met. ANOVA was used to test for differences between groups in OSCE 1, OSCE 2 and OSCE 3 outcomes. Repeated measures of ANOVA were used to ensure the effectiveness of the intervention in each group. The OSCE test results were given for each station to compare the results of the traditional teaching method with the game teaching method. The results of the cardiovascular surgery, gastroenterology and thoracic surgery stations in group A and the ENT, neurology and orthopaedic surgery stations in group B, where the surgical sets were presented in the form of game, were combined for analysis as game-based teaching. Group B stations (cardiovascular surgery, gastroenterology and thoracic surgery) and group A stations (ENT, neurology and orthopaedics), where surgical sets were routinely provided, were recorded and reported as traditional teaching.

## Results

The present study aimed to investigate the effects of gamified surgical sets on students’ ability to arrange surgical sets in the operating room. According to the Shapiro-Wilk test, the data collected had a normal distribution, and no outliers were found. The pretest scores of students in groups A, B and C were not significantly different (*P*-value (95.00% CI of diff.)); (A vs B, 0.907 (-1.559 to 1.092), A vs C, 0.937 (-1.125 to 1.525) and B vs C, 0.716 (-0.8920 to 1.759)). For each of the six OSCE test stations (1, 2 and 3) in each group, the mean results are shown in Table 2. The results for the groups with game-based instruction and routine training are highlighted.

### Comparing game-based and traditional teaching

The one-way ANOVA revealed that the scores between groups A and B were not significantly different in terms of performance in a non-clinical setting (OSCE 1), a clinical setting (OSCE 2), and 3 months after the intervention (OSCE 3). However, when comparing the two teaching methods, there was a significant difference in scores for all three assessments (Table 3). Specifically, game-based instruction outperformed traditional teaching, with statistical significance observed for OSCE 1 in particular, where game-based teaching displayed markedly higher levels of performance compared to traditional instruction (*P* < 0.0001). The study revealed significant differences in the effectiveness of game-based and traditional teaching when comparing OSCE 2 results in a clinical setting. Furthermore, comparing the results of OSCE 3 3 months after the intervention, it became evident that game-based instruction demonstrated longer-term sustainability in promoting learning retention compared to traditional instruction.

**Table 3:**
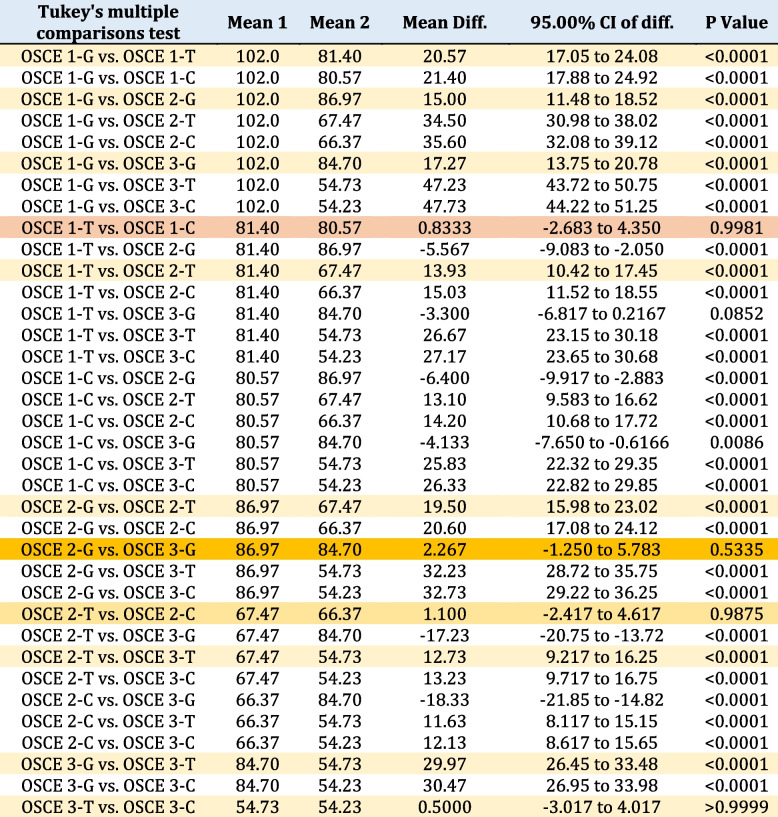
Comparison of Tukey post-hoc OSCE test scores in three groups: game-based learning, conventional training and the control group

The study revealed significant differences in the results of game-based teaching compared to traditional teaching (OSCE 1 and OSCE 2). The results from OSCE 2 were highly significant (*P* < 0.0001) when comparing the two methods. Interestingly, there was no significant difference observed in students’ mean scores between OSCE 2 and OSCE 3 for game-based instruction (*P* = 0.5335). However, there was a notable decrease in students’ mean scores for OSCE 3 in the traditional teaching group, indicating a decline in performance (*P* < 0.0001). Additionally, comparing mean scores between OSCE 1 and OSCE 3 showed considerable decreases within the traditional group (*P* < 0.0001), according to Table 3. Furthermore, upon comparison with the control group’s results, it was found that their performance remained similar without any statistically significant differences identified. Nevertheless, it is important to note that, like the traditional teaching method’s outcomes, there existed a noteworthy decline in participants’ performances during their evaluation through the initial examination (OSCE 1) or another following the final stage (OSCE 3), emphasizing similarity among these different approaches (Table 3).

### Assessment of effectiveness intervention

**Fig. 5:**
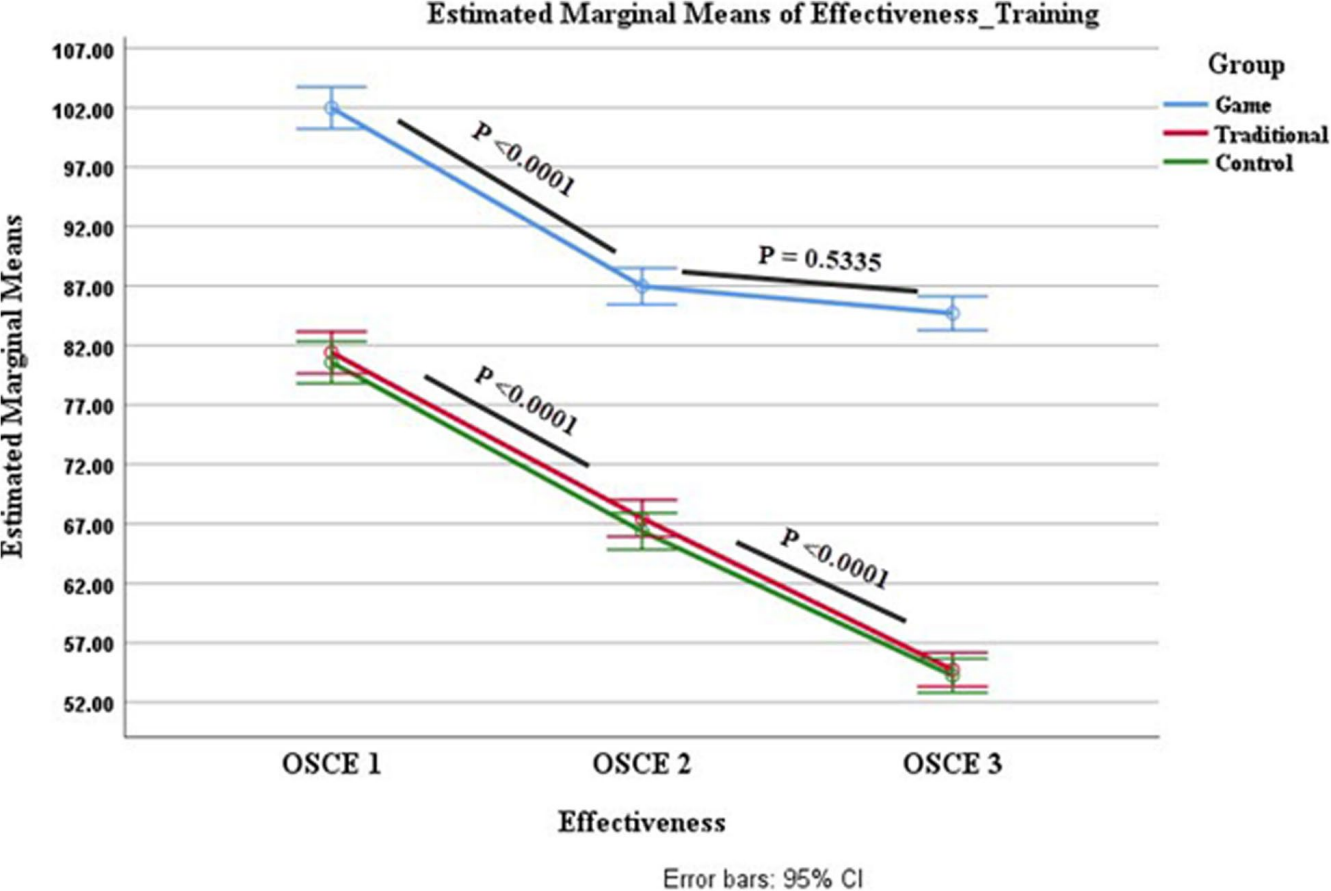
Repeated measures tests were used to measure the effectiveness of training in each group on OSCE outcomes. Compared to the traditional training group, the game-based learning group was more effective, and students achieved significant results

Analysis of variance with repeated measures showed that the teaching methods in game-based learning significantly improved the participants’ ability to distinguish surgical instruments. There was also a noticeable decline in performance in the traditional teaching method and the control group (Fig. 5).

## Discussion

Crossover design is an experimental method in education where participants are assigned to two or more approaches in a sequential order, aiming to balance the effects of individual differences [23]. It’s useful for studying short-term or reversible interventions and also when a control group isn’t feasible. However, it has limitations like carryover and period effects, necessitating careful planning and analysis for validity and reliability [24]. Several studies have delved into the intricacies of crossover designs in education research. One such study conducted by Lewis in 1998 thoroughly explores the construction of these designs, specifically focusing on balancing the effects of carry-over from unrelated factors [24]. Additionally, Zhou’s investigation in 2012 offers valuable insights into analyzing data from crossover designs using linear mixed-effects models. This comprehensive analysis considers various factors such as treatment effects, period effects, and carry-over effects [25]. In a similar vein, Parienti introduces an innovative cluster-crossover design approach in their 2007 study, which stresses allocating treatment sequences at the cluster level and emphasizes the importance of hierarchical models for accurate data analysis [23]. Moreover, Wellek’s work published in 2012 underscores that to guarantee scientific validity, it is crucial to analyze results separately based on sequence group while also considering any potential carry-over effects present within crossover trials [26].

The crossover design implemented in this study allowed for exposure to the game by students in both groups, thereby ensuring unbiased and dependable results. Notably, the findings from OSCE 1 and OSCE 2 within the traditional teaching group revealed that clinical conditions significantly impacted students’ performance accuracy. However, when we compared these two assessments within the game-based teaching group, no noteworthy differences emerged. These results suggest that game-based learning effectively sustains student accuracy under stressful circumstances without compromising performance levels. A plethora of studies have consistently demonstrated the positive impact of game-based instruction on learning outcomes [27, 28]. Furthermore, the results of the study revealed a significant reduction in the time taken by game-trained students to set up surgical instruments compared to conventionally trained students. These findings support the hypothesis that incorporating gaming elements can enhance performance in arranging surgical instruments among surgical technology student novices. One possible explanation for this improvement is that the game exposes users to various scenarios and provides effective feedback, allowing them to identify and rectify errors. Additionally, comparing the results from OSCE 3 with those from OSCE 2 reveals that learning through games can also improve performance durability.

The effects of game-based training in surgical instrumentation had been researched in the past, mainly with medical students and surgical residents [29–31]. The study conducted by David B. Clarke with first-year neurosurgery residents showed that recognition of surgical instruments improved with repetition when using the PeriopSim™ platform. Trainees showed a significant increase in overall scores and time saved, as well as a reduction in the number of errors over three testing sessions [32]. In the study conducted by Paim CPP, an educational game for placing surgical instruments on the mayo stand or back table was developed as a tool to assist the instructor in teaching surgical instruments to students and nurses in further education - a computer-based game derived from a Portuguese game called “Playing with Tweezers” [33]. This game was validated in Morteza Nasiri’s study, the English version was tuned in two successive steps, and the final round, “Playing with Surgical Instruments (PlaSurIn)”, was developed [34]. Recently, the effectiveness of the PlaSurIn game was studied by operating room students, and it was found that the knowledge and performance of operating room novices improved with this game [35]. It is worth noting that the present study had the following advantages: (1) It used a framework that the authors had proposed as a basis for developing the game. The game’s development was based on the native language, and face and content validity were also investigated. (2) Three groups from two institutions were studied. (3) Student performance was assessed at two sites (non-clinical and clinical), as well as learning retention. (4) The effectiveness of the training programme was also assessed through repeated measures testing. (5) This study was also designed as a crossover study so that two intervention groups could participate in the game, ensuring unbiased and dependable results.

The design of games is critical to the effectiveness of games as learning tools. Educative games should consider current and future conditions because, with this insight, they can keep their users engaged and bring about lasting change. However, user empowerment is an important issue in game-based education. In the traditional education system, there is more emphasis on teamwork than individual collaboration, but who should make the final decision? Students must understand that they must rely on themselves in the workplace and the hospital and take responsibility for their actions. A study conducted on nursing students’ decision-making in crisis situations has shown that teaching through games can lead to behavioural fluency [9, 36]. Three features characterise a concept of behavioural fluency: Durability (continuity over time), Stability (i.e. maintenance of behaviour in a distracting environment) and Generalisability (generalisation to a new environment) [37–39]. In this context, it would be appropriate to consider this concept equivalent to the third level of Kirkpatrick’s pyramid, which aims to influence learners’ behaviour in the workplace. Therefore, games can meet the needs of this group of students. In other words, it is advisable to keep in mind the primary goals of education before starting to develop intervention strategies.

The crossover design allows for a comprehensive evaluation of the effectiveness of an intervention throughout different stages. A recent study conducted by Masoumian et al. using a crossover design demonstrated that incorporating games at the outset of teaching and coupling it with teacher training as instruction progresses can lead to meaningful learning outcomes. This positive impact may be attributed to creating a welcoming and engaging environment at the start of each new class. Thus, while games are valuable in enhancing education, their sole reliance cannot guarantee meaningful learning experiences. However, considering course content and integrating games strategically as educational prerequisites can yield significant results [40]. Moreover, completely transitioning from traditional education to solely technology-based approaches remains impractical currently; instead, merging technology with conventional teaching methods emerges as an ideal approach to foster profound educational journeys [41].

## Conclusion

This study used a crossover design to implement game-based learning in two groups. Furthermore, the learning assessment was based on the OSCE tests, which have high validity. In addition to the pedagogical design of the game, the availability of the game also contributed to the improvement of students’ skills and the durability of their performance. From the study results, this game can improve the training of specialist students, which is essential for reducing surgical errors.

### Study implications

Considering the results, the Surgical Instrument Arrangement Game could be used as a suitable alternative or supplement to traditional training methods to develop students’ surgical instrument arrangement skills. As this game is free and has a straightforward platform, it can be integrated into future training programmes to improve the knowledge and performance of operating room novice surgical instrument arrangers in limited time and with limited educational resources, especially during COVID -19 downtime when routine training methods are not available.

These results can be applied to other universities with similar demographic characteristics. In addition, the game could be used to arrange surgical instruments to train medical students during their residency training in operating rooms, e.g. inexperienced nurses and surgical residents. However, the generalisability of the present findings to other students with a health-related major requires further investigation.

One crucial aspect to consider when examining the generalizability of data is implementing a crossover design in educational studies. One major challenge with this approach is the information leakage between two groups, posing a threat to internal validity. To mitigate this bias, we used two nearly identical institutions in our study. By doing so, we ensured that both groups were unaware of each other’s existence, thus enhancing the reliability and credibility of our results. In addition to the factors mentioned above, it is important to consider the applicability of these results across different student demographics. Cultural relevance, prior educational background, access to technology, language proficiency, and level of support provided during the game can all influence engagement levels and impact learning outcomes and performance in OSCEs.

### Study strengths

As far as we know, this study was the first intervention study to evaluate an educational game on surgical instrument placement in surgical technology students. This study was notable because it evaluated performance and retention. The OSCE procedure was used because it has been shown to be a well-validated assessment method [42]. This study stood out due to its meticulous attention to pedagogical design, as the game’s development was rooted in a well-constructed framework that aligned with specific learning objectives. The implementation process also followed these predetermined steps, ensuring a comprehensive and unbiased approach. Furthermore, the crossover design employed in this study facilitated equal access for all students in the intervention group, thereby yielding reliable results.

### Limitation

We could not include the effects of stress from the clinical setting in the pretest because it was conducted in a non-clinical setting. However, as the pre-test was the same for both groups, it is unlikely that this had a negative impact on the intervention. In addition, all students were influenced by the game and traditional teaching, so the grouping did not affect the results.

## Data Availability

Upon a reasonable request, the corresponding author can provide the data set that was analyzed during this study.
